# Using minimal human-computer interfaces for studying the interactive development of social awareness

**DOI:** 10.3389/fpsyg.2014.01061

**Published:** 2014-09-26

**Authors:** Tom Froese, Hiroyuki Iizuka, Takashi Ikegami

**Affiliations:** ^1^Departamento de Ciencias de la Computación, Instituto de Investigaciones en Matemáticas Aplicadas y en Sistemas, Universidad Nacional Autónoma de México, Ciudad UniversitariaMexico; ^2^Centro de Ciencias de la Complejidad, Universidad Nacional Autónoma de México, Ciudad UniversitariaMexico; ^3^Laboratory of Autonomous Systems Engineering, Graduate School of Information Science and Technology, Hokkaido UniversityHokkaido, Japan; ^4^Ikegami Laboratory, Department of General Systems Studies, Graduate School of Arts and Sciences, University of TokyoTokyo, Japan

**Keywords:** social cognition, joint action, social interaction, intersubjectivity, second-person perspective, consciousness, developmental psychology

## Abstract

According to the enactive approach to cognitive science, perception is essentially a skillful engagement with the world. Learning how to engage via a human-computer interface (HCI) can therefore be taken as an instance of developing a new mode of experiencing. Similarly, social perception is theorized to be primarily constituted by skillful engagement between people, which implies that it is possible to investigate the origins and development of social awareness using multi-user HCIs. We analyzed the trial-by-trial objective and subjective changes in sociality that took place during a perceptual crossing experiment in which embodied interaction between pairs of adults was mediated over a minimalist haptic HCI. Since that study required participants to implicitly relearn how to mutually engage so as to perceive each other's presence, we hypothesized that there would be indications that the initial developmental stages of social awareness were recapitulated. Preliminary results reveal that, despite the lack of explicit feedback about task performance, there was a trend for the clarity of social awareness to increase over time. We discuss the methodological challenges involved in evaluating whether this trend was characterized by distinct developmental stages of objective behavior and subjective experience.

## Introduction

Theories about the primacy of embodied interaction over detached social cognition have grown in popularity. For example, there are interaction theory and the narrative practice hypothesis (Gallagher and Hutto, 2008), the concepts of participatory sense-making (De Jaegher and Di Paolo, 2007) and self-other co-determination (Thompson, 2001), the formal methods of interpersonal synergies (Riley et al., 2011) and social coordination dynamics (Oullier and Kelso, 2009), and the second-person approach to neuroscience (Schilbach et al., 2013). Closely related to this emphasis on embodiment and social interaction is the hypothesis of direct perception of other minds (Gallagher, 2008a; Krueger, 2012; Stout, 2012), which holds that perceptual social experience normally takes precedence over, and provides the concrete basis for, reflective social cognition such as simulating and theorizing. These theories thereby doubly break with psychology's traditional emphasis on an individual's thinking as the essential basis of social awareness (e.g., Wegner and Giuliano, 1982).

These theories, which accord primacy to social perceptual interaction in adult social cognition, are naturally complemented by theories that accord primacy to this social perceptual interaction in the development of social cognition in infancy (Gallagher, 2008b). For example, preverbal infants' understanding of other minds is argued to originate and develop within mutual engagement (Reddy and Morris, 2004), second-person interaction (Fuchs, 2013), and primary intersubjectivity (Trevarthen, 1979). Within this context of co-regulated activity an infant's intentions can emerge and be realized in joint action (Fogel, 1993). Again, this kind of embodied interaction is not conceived as a purely unconscious phenomenon, since an infant's movement always already implies a certain form of animation and affectivity (Sheets-Johnstone, 1999). Rather, embodied interaction is seen as going hand in hand with the development of what has been called dyadic states of consciousness (Tronick, 2004) and self-other conscious affect (Reddy, 2003). A similar emphasis on the developmental precedence of communal embodied coupling before self-other differentiation can be found in the phenomenological psychology of Merleau-Ponty (1964). By extending his account to prenatal development, it can even be argued that the maternal body and fetal body are already situated in an embodied interaction that is affectively structured through the negotiated movements themselves (Lymer, 2011).

The primacy of embodied-social-perceptual interaction is therefore supported by a variety of empirical and theoretical traditions that are progressively being integrated into a cohesive research program (Froese and Gallagher, 2012). However, while this emerging framework is compelling for many, from the mainstream perspective it still needs to further prove its worth compared to the traditional framework by making unique predictions that are experimentally verified.

Following the enactive approach, we have recently provided evidence for the interactive constitution of intersubjective awareness in pairs of adults using a minimal haptic human-computer interface (HCI) (Froese et al., 2014), namely an experimental setup which is known as the “perceptual crossing paradigm” (Auvray and Rohde, 2012). Subsequently, given the close theoretical link between interactive approaches in cognitive science and developmental psychology, we hypothesized that the same kind of setup could also provide insights into the early development of intersubjective awareness. Promisingly, related research with pairs of interacting adults has shown that it is indeed possible to study the development of new communication systems (Galantucci and Garrod, 2011), including on the basis of purely embodied interactions (Iizuka et al., 2013). We were therefore interested to determine whether some preliminary evidence to support this hypothesis could be found by extending the analysis of our original experiment to include diachronic aspects of the interaction process. Given a trial-by-trial analysis, would we find indications of a sequence of developmental stages of social awareness, such as those proposed by interaction-oriented developmental psychologists? We derived the specific form of our hypothesis on the basis of the following considerations.

It has been argued that the phenomenal quality of perception is largely constituted by the specific dynamical form of its underlying sensorimotor skill, rather than just by a dedicated biological organ and/or neural system (e.g., O'Regan and Noë, 2001; Noë, 2004; Mcgann, 2010). Moreover, it follows that if perceptual experience is indeed constituted by skillful sensorimotor interaction then incorporating some form of *mediation* into that embodied interaction will result in a corresponding *modulation* of that experience. Learning how to practically engage the world via new tools, HCI, and other mediating systems^1^ is associated with the emergence of new ways of being in and experiencing the world, that is, technology is conceived as anthropologically constitutive (Havelange, 2010). Some modulations are relatively subtle changes in perceptual experience (e.g., Davoli et al., 2012), while other phenomenological changes, such as those induced by one's mastery of sensory substitution systems, can be more profound (Lenay et al., 2003; Auvray and Myin, 2009). As we have observed in our research with various kinds of HCIs, the fact that skillful usage of an HCI must first be learned provides us with an opportunity to systematically investigate the development of new modes of perceptual experiencing (Froese et al., 2012). An added methodological bonus is that this development can happen long after infancy, i.e., at a time when the typical adult participants' standard perceptual modalities have normally long been formed already. This idea that learning can recapitulate ontogeny is supported by a tradition in psychology centered on the “microgenetic” method, which has also observed that older individuals sometimes regress to the strategies and developmental trajectories of younger individuals when they are learning an unfamiliar task (Miller and Coyle, 1999).

We were therefore led to the following hypothesis: if we accept the enactive theory that social experience is constituted by skillful interactions with others (Mcgann and De Jaegher, 2009; Froese and Di Paolo, 2011), and our proposal that learning how to co-regulate a mutual interaction by means of an unfamiliar HCI is tantamount to re-acquiring such a social skill, then our original perceptual crossing study should have provided the conditions for the recapitulation of typical infants' developmental stages of social awareness during repeated embodied interaction by pairs of adults. In order to determine whether this hypothesis is worthy of further systematic consideration, we re-analyzed the objective and subjective data from our original study in a diachronic manner. Given the post hoc nature of this analysis, the results are only preliminary. And, while they already look promising in some respects, they also serve to highlight areas where more methodological fine-tuning is still needed.

## Theory and methods

Using technological interfaces has been indispensable for providing support for an interactive approach to social development. For example, evidence for Trevarthen's (1979) notion of primary intersubjectivity has been obtained on the basis of his double TV monitor paradigm, which allowed the insertion of recorded video footage into a live face-to-face interaction (Murray and Trevarthen, 1985). Using this kind of setup, it has repeatedly been demonstrated that infants are sensitive to the co-regulation of social interaction (Nadel et al., 1999). Although cognitivist interpretations of these findings are possible, agent-based modeling of Trevarthen's experimental paradigm has contributed to the formalization of this sensitivity in terms of dynamical systems theory (Di Paolo et al., 2008). And while such modeling can lend formal support to a phenomenological analysis of the structures of intersubjectivity (Froese and Fuchs, 2012), what we are still lacking is an experimental paradigm that allows researchers to systematically investigate the development of social awareness as it is experienced from the first-person (or second-person) perspective.

Indeed, the scientific study of the development of social awareness is confronted by serious methodological challenges. Only in the last decades has there been a growing appreciation of infant consciousness (Trevarthen and Reddy, 2007), and their social experience has been investigated from the second-person perspective, that is, based on the concrete experiences of developmental psychologists who frequently interact with infants (Reddy, 2003; Reddy and Morris, 2004; Tronick, 2004). Clearly theories about infant phenomenology devised through such engagement are valuable, but it would still be desirable to verify them from the infant's perspective. However, in the absence of verbal skills it is difficult if not impossible to apply the usual first- and second-person methods used in the science of consciousness (e.g., Froese et al., 2011). And while adult investigations of the phenomenology of intersubjectivity provide detailed insights into how we experience others (Ratcliffe, 2007), adults take social awareness for granted and can no longer remember how it had originally developed^2^.

**Figure 1:**
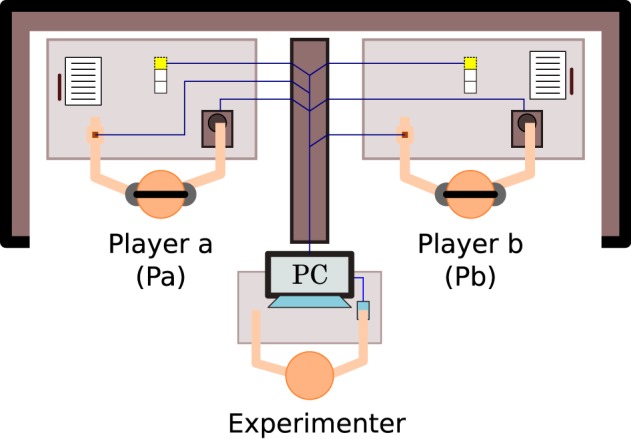
**Experimental setup of perceptual crossing paradigm**. The two participants can only engage with each other via a human-computer interface that reduces their scope for embodied interaction to a bare minimum of translational movement and tactile sensation. Each player's interface consists of two parts: a trackball mouse that controls the linear displacement of their virtual avatar, and a hand-held haptic feedback device that vibrates at constant frequency for as long as the avatar overlaps with another virtual object and remains off otherwise. Three small lights on each desk signal the start, halftime (30 s), and completion of each 1-min trial. Figure adapted from Froese et al. (2014).

**Figure 2:**
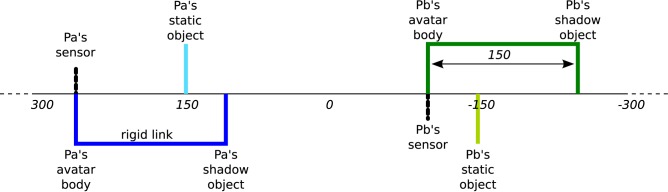
**Virtual environment of perceptual crossing paradigm**. Players are virtually embodied as “avatars” on an invisible line that wraps around after 600 units of space. Each avatar consists of a binary contact sensor and a body object. Unbeknownst to the players a “shadow” object is attached to each avatar body at a fixed distance of 150 units. There are also two static objects, one for each player. All objects are 4 units long and can therefore only be distinguished interactively in terms of their different affordances for engagement. Figure adapted from Froese et al. (2014).

To overcome this problem we took advantage of the perceptual crossing paradigm in psychology (Auvray et al., 2009), which has enabled researchers to systematically investigate the real-time self-organizing dynamics of dyadic interaction by mediating embodied interactions of pairs of adults over a minimal HCI (Figure 1). Participants are embodied as avatars in a 1D virtual environment (Figure 2). They can move their avatar left and right, and they receive haptic feedback in the form of a constant vibration to their hand for as long as their avatar overlaps with any other virtual object (otherwise the feedback remains turned off). Each participant can encounter three objects: their partner's avatar, an exact copy of the other's avatar that moves at a constant distance from the avatar (which we call the “shadow” object), and a simple static object (one for each player at distinct locations). All objects have the same size and provide the same haptic feedback. They can only be distinguished by means of their differing affordances for interaction.

Participants are instructed to click in order to signal to the experimenters when they have recognized that the object, with which they have been interacting, is their partner's avatar. Participants cannot directly perceive each other's clicks and no feedback is provided until after the experiment. In other words, in order to establish an embodied communication system they must learn how to distinguish between sensations that are generated by their own actions from those generated by the movement of external objects (the problem of separating self from non-self), and to distinguish external movements that express a communicative intention from those that do not. The latter challenge not only involves finding some responsive object as such (the problem of detecting social contingency), but also learning how to differentiate between movements made to change location and movements made with specific communicative intent (the problem of signaling signalhood). And since there is no kind of external feedback, learning can only be guided by impressions obtained via these interactions themselves. It is a formidable task indeed.

Methodologically, this kind of experimental approach shares notable similarities with the microgenetic method of developmental psychology (Siegler and Crowley, 1991). According to Rosenthal (2004), the latter draws on a long tradition which had two key methodological aims, namely “to provide the means of externalizing the course of brief perceptual, or other cognitive processes by artificially eliciting ‘primitive’ (i.e., developmentally early) responses that are normally occulted by the final experience” and “to construct small-scale, *living models* of large-scale developmental processes in such a way as to ‘miniaturize’ (i.e., accelerate and/or telescope) the course of a given process and bring it under experimental control” (p. 221). Regarding the perceptual crossing paradigm, the choice of asking participants to interact via a novel, minimalist HCI is motivated by the same aims of externalizing and recapitulating the processes underlying the constitution of otherwise already formed perceptual experiences, and thereby making these processes available for scientific investigation (for another example of this approach, see Lenay and Steiner, 2010). Although it could be argued that these methods are confusing development with learning, the distinction between these processes of individual change is not that clear-cut. In addition, the hypothesis that the processes underlying changes that are occurring on differing time scales share important commonalities has long been a useful working hypothesis in developmental psychology ^3^.

In the original experiment by Auvray et al. (2009), as well as in several subsequent variations (for a review, see Auvray and Rohde, 2012), it was found that differences in the relative stability of interactions ensured that participants managed to locate each other while avoiding the shadow and static objects. Interaction with the static object is too stable and predictable to be human, whereas the shadow object is too unstable since it moves but does not respond; only the other's avatar can respond to contact by reacting and sticking around. Yet this interactive self-organization of a situation of mutual tactile interaction apparently did not generally coincide with the emergence of an individual awareness of the actual presence of the other participant. While participants signaled recognition more frequently during mutual interaction, thereby objectively solving the task, this could also have simply been a statistical consequence of the fact that they spent more time in mutual interaction. Importantly, Auvray et al.'s statistical analysis revealed that the probability of clicking was not significantly higher after making contact with the other player when compared with its unresponsive shadow copy. Although it is possible that participants were genuinely aware of having engaged with their responsive partner in some cases, this could not be shown with the data. The results therefore fell short of conclusively demonstrating an interactive constitution of social cognition, where social cognition is conceived as resulting in a personal-level insight (Michael and Overgaard, 2012).

On the basis of agent-based models and theoretical considerations, Froese and Di Paolo (2011) hypothesized that this lack of personal recognition of the other was to be expected given that the experimental task was not genuinely social, at least if the mark of the social is conceived specifically as the co-regulation of mutual interaction. Through their coupled behavior the pairs of participants in these studies were forming a multi-agent system of sorts, but without any additional incentive to engage in co-regulated joint action there was little opportunity for social experience, and alongside it individual recognition of the other's presence, to emerge consistently. The original task of clicking whenever encountering the other also allowed purely individualistic strategies to be successful. For example, simply waiting until an object repeatedly made contact, which indicates that it must be the other because she is sensitive to one's presence as an object in the virtual space, and then clicking. However, from the searching other's perspective this kind of unresponsive strategy makes it impossible to distinguish the partner as such. For a genuinely social, that is, shared situation to emerge there has to be mutual engagement.

Froese et al. (2014) tested this hypothesis by running a perceptual crossing experiment in which participants formed teams in a tournament game and were explicitly instructed to help each other with the task of locating each other. In this study 17 pairs of adults completed a sequence of 15 one-minute trials. For each trial they were asked to click once (and once only) as soon as they became aware of the other player's presence. After each trial in which a participant had clicked they were asked to rate the clarity of their experience of their partner on a Perceptual Awareness Scale (PAS) that was adapted for this purpose from Ramsøy and Overgaard (2004), and to give a short free-text description of that experience and their strategy. Specifically, players were asked to give a PAS rating between 1 and 4: “Please select a category to describe how clearly you experienced your partner at the time you clicked: (1) No experience, (2) Vague impression, (3) Almost clear experience, (4) Clear experience.” The hypothesis was confirmed: clicks were significantly more probable after contact with the other, most trials led to accurate identification of each other, and such joint success was correlated with high ratings of clarity of the other's presence.

Although that study was not designed to specifically investigate the development of social awareness, our interest in conducting such a diachronic analysis of the results was provoked by some of the first-person reports provided by the participants. As we expected, there were many reports describing forms of joint attention and joint action, for example turn-taking and imitation. Surprisingly, however, there were also quite a few individual-centered reports in which participants described their experience of the other's presence in terms of the other's actions toward themselves. This is a specific kind of second-person awareness that is familiar from the developmental psychology literature. Reddy (2003) has argued that social awareness in the first couple of months in an infant's life primarily consists in being the object of the other's attention, while more advanced forms of mutual attention, including joint attention on aspects of the social interaction itself, develop in subsequent months. In retrospect this finding of a possible recapitulation of the development of social awareness is not that surprising; it follows quite naturally from enactive theories of perception and social interaction, as we argued in the introduction.

## Diachronic analysis and results

In the following we present a diachronic analysis of the perceptual crossing study first described in Froese et al. (2014). First, we were interested to determine if there was an effect of implicit learning in terms of changes in the objective results. Although participants were not given any information about the success of their clicks during the experiment, there might still have been tendencies toward improvement over the sequence of trials. Second, we wanted to see if we could find any qualitative transformation of user experience over trials, both in terms of PAS ratings and in the brief first-person reports written by participants.

### Evidence for implicit learning

Right at the beginning of the diachronic analysis we noticed that there was a potential confounding factor in the way we had designed the original study. Although we had randomized the starting configurations of the 15 trials, we had neglected to randomize the starting configurations across teams. This makes no difference if we are interested in aggregate performance only (like in the original study). However, when analyzing performance over trials, there is a possibility that trends in the results were influenced by accidental trends in the starting positions, in particular players' initial distance to each other and to their static objects. Although such influence cannot be ruled out in principle, we did several tests and did not find any compelling dependency on starting positions (for details see Supplementary Information, Section S1). It is likely that the 60 s available during each trial were sufficient for starting positions to be of little influence with regard to the final outcome.

**Figure 3:**
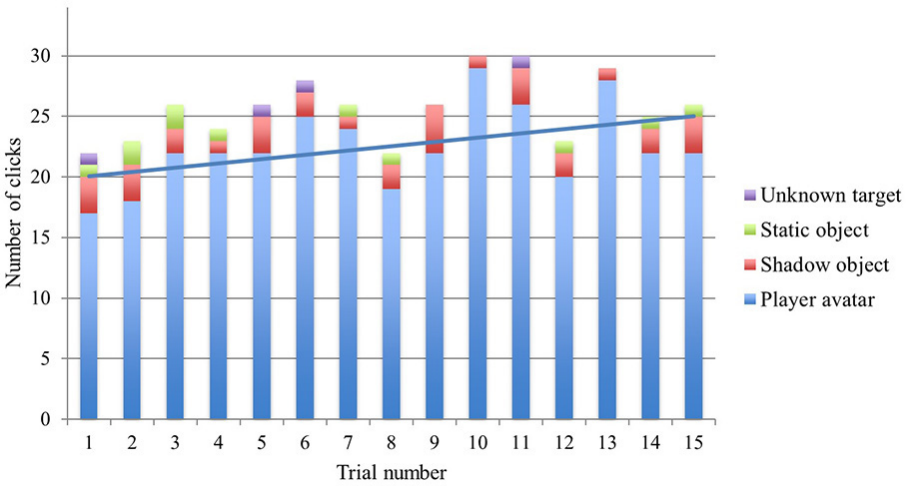
**Cumulative column chart showing changes in the number of clicks on different targets over the 15 trials**. The maximum number of possible clicks per trial number is 34 (2 players × 17 teams). The linear trend line refers to avatar clicks only. For details of how the different virtual objects were determined to be the target of a click, see the methods section in Froese et al. (2014).

As a first step toward detecting the effects of implicit learning we can consider how the frequencies of clicks on object types changes over the 15 trials (Figure 3). During the first half of trials there is a consistent tendency toward an increasing number of clicks on the other's avatar. This upward trend generally continues during the second half of trials. Three of them result in the three highest number of avatar clicks (i.e., trials 10, 11, and 13). But there is also a notable lack of consistency: all of the other later trials resulted in notably less avatar clicks, although never less than the very first couple of trials. Interestingly, in most cases these later reduced successes cannot be explained by corresponding increases in wrong clicks. Rather, it is the total number of clicks that is temporarily decreased (see especially trials 8 and 12). In other words, these later fluctuations seem to be partially the result of more conservative choices: the players seem to have implicitly learned how to identify their partner, but perhaps the opportunity to do so did not present itself clearly enough in those trials to warrant a click ^4^. Nevertheless, even so it remains an open question why these later moments of increased uncertainty consistently arose across the 17 teams.

Stewart (2010) has noted that when we are talking with others we tend to give them the benefit of the doubt that any uncertainties we may have about what they meant to say will be resolved as our interaction proceeds. After a while we may become active participants in this process of resolution by asking: “what did you mean when you said that… ?” The temporary decreases in avatar clicks may thus reflect attempts to gain more certainty by renegotiating the interaction process. Cuffari (2014) has argued that jointly overcoming breakdowns of sense-making is intrinsic to the emergence of shared meaning. On the basis of the first-person reports we can see that something similar is going on here in some cases, as forms of co-regulation emerge, stabilize, become questioned, and dissolve again.

For example, in one session (experiment 18) two players were trying to co-create a shared signal. After trial 3 player “b” wrote: “I collided with a moving object but the first and second periods of the appeal were different so I recognized it was the simple moving object and searched again” (E18T3Pb)^5^. Eventually the players reached an agreement about the shape of their signal, which is why the same player wrote self-assuredly after trial 9: “Receiving and sending. Do either role alternately” (E18T9Pb). However, later on doubts about whether a meaningful connection had actually been established start to creep in. After trial 14 the player explained: “Appeal and wait. But the object that I touched generates clear three-times-signal with constant period and it happens twice. So I did not click because I felt it was so mechanical” (E18T14Pb).

**Figure 4:**
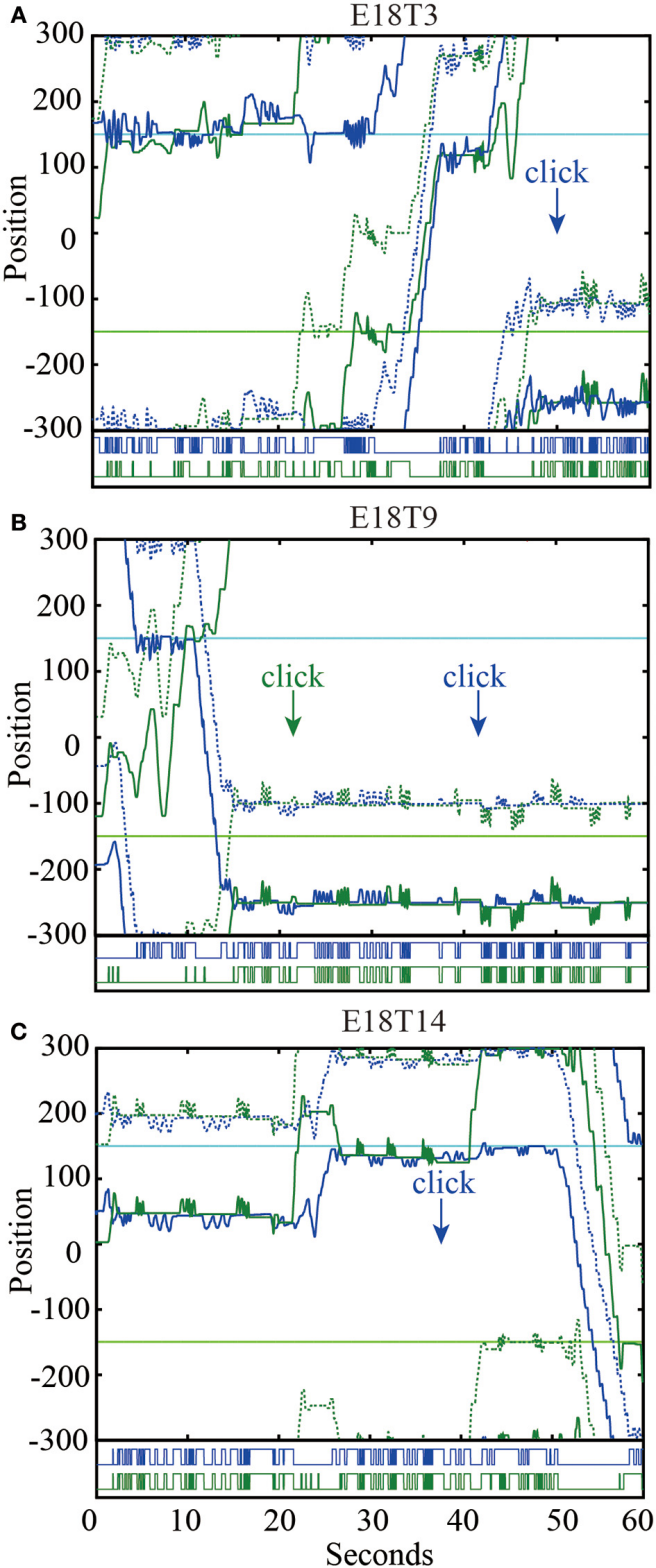
**Virtual trajectories over 60 s of three representative trials**. Player a (Pa) is shown as blue, while player b (Pb) is green (see Figure 2). Solid and dashed lines represent positions of avatar and shadow objects, respectively. Light blue and light green solid lines show the positions of the static objects detectable by Pa and Pb, respectively. The bottom of each plot shows the haptic feedback (on/off) received by each player. **(A)** In trial 3 players find each other quickly, but Pb can be seen to break off their interaction. At no point is Pb interacting with the shadow object (a “simple moving object”), but the unexpected irregularity of responses he describes could be attributed to interference caused by Pa's static object. **(B)** Trial 9 begins with some difficulties as Pb briefly interacts with Pa's shadow object and Pa becomes distracted by his static object. Eventually they find each other and start “receiving and sending” tactile stimuli while adopting either role alternately. Note that their exchanged activity consists of varying frequencies and durations. **(C)** In trial 14 we see two periods of turn-taking activity. In both cases Pa keeps sending a slow and regular “three-times-signal” while Pb's activity is faster and more irregular. Both times Pb abruptly departs from the interaction after a few exchanges, thus explaining why Pa is left feeling that “the partner ran away during the trial.”

It is interesting to note the shifting conditions of communication: the same player who earlier on rejected an interaction because the repeated appeal was too “different” later ends up rejecting an interaction because the already established appeal was repeated “twice.” Of course, the other player noticed that the signal failed to elicit the desired response: “I could not get the good response. I felt that the partner ran away during the trial” (E18T14Pa), and is left wondering about the reasons for this breakdown: “I felt that there was an interruption while communicating. It might be because a very fast object passed or I made a mistake” (E18T14Pa). Although it could be debated whether we can trust subjects to report accurately about their experience and about what is objectively going on (Jack and Roepstorff, 2003), here we decided to give participants the benefit of the doubt. There is no reason to assume that their reports are systematically misleading; see, e.g., Figure 4.

We discussed this example at length because it serves to show the complexity of the development of human communication. We should not expect to find a linear or even smooth developmental progress, since we are not dealing with machine learning like a hill climbing algorithm. If there had been further trials, this pair might have resolved their crisis and established another communication system with renewed, and perhaps even increased, confidence. For example, they could have meaningfully incorporated that repetition of the “three-times-signal” or even dropped it altogether. There is no reason to assume interactive alignment or convergence of behaviors, because progress in a coordinated dialogue requires differentiation of interlocutors' turns (Mills, 2014). Too much repetitive imitation may be interpreted as a failure to communicate, as we saw in this example. Both the means and goals of social interaction change over time, and these dialogical changes can go beyond the intentions of the individuals (Fusaroli et al., 2014). Relatedly, two common findings of microgenetic studies of learning, which are consistent with our analysis, are the halting and uneven use of newly acquired competencies and, more surprisingly, that changes in strategies are also often initiated following successes rather than just failures (Siegler and Crowley, 1991).

**Figure 5:**
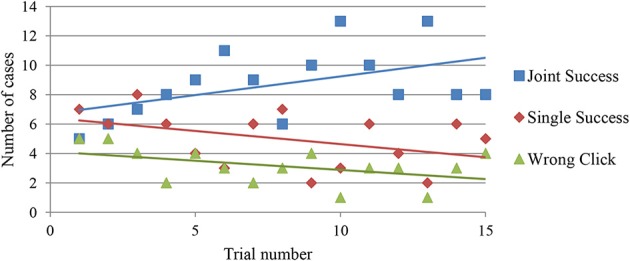
**Changes in number of different types of clicking situations over the sequence of 15 trials**. “Joint Success” shows the number of trials where both players clicked correctly, and “Single Success” shows the number of trials where one player clicked correctly while the other player clicked wrongly or not at all. “Wrong Click” shows the number of wrong clicks.

Another way of measuring implicit learning is by evaluating whether the amount of co-regulated activity changed over trials. For example, clicking success may come from a lucky guess, it may be the result of an individualist strategy such as waiting for the “prey” to trigger the sensor without moving oneself, or it may be the outcome of reciprocal interaction and joint action. While it is difficult to objectively differentiate between the various possibilities, a useful heuristic is to at least distinguish between trials in which both players were able to click successfully (“Joint Success”) from trials were only one of the players clicked successfully (“Single Success”). And both of these cases can be contrasted with clicks that were simply wrong (“Wrong Click”)^6^. Figure 5 shows how the number of each of these three categories changed over the sequence of 15 trials. It reveals that there is a tendency for trials with jointly correct clicks to increase in frequency.

This tendency toward more Joint Success could be a sign that the players were able to develop better ways of mutually identifying each other. However, arguably it could simply be contingent on an increase in successful individualistic strategies, because more individual successes would independently add up to more cases in which both players click successfully, even if they did not directly facilitate each other. Yet while this could be the explanation of some cases of Joint Success, it is unlikely to be the whole story because a strategy of trying to detect the other without actively making oneself detectable to the other is less likely to lead to Joint Success.

An indication of the co-dependence of correct clicks can be gained by analyzing their temporal relationship within a trial. At first sight the delays between jointly successful clicks support a more interactive interpretation of the results. In most trials where both players correctly clicked on the other, they did so within seconds of one another (23% co-occurred within 3 s), which suggests that we are dealing with cases of mutual attention that led to mutual clicking (see Figure 4 in Froese et al., 2014). Yet when we look at the distribution of clicking delays over the sequence of trials (Figure S5), the picture becomes more complex: the increase in the number of Joint Success trials is largely due to an increase in Joint Success trials with mutual clicking delays longer than 10 s. Presumably this is because participants have developed the capacity for more sustained interactions, thus eliminating the need to click as soon as possible when detecting the other's presence. The interaction process may also have become an interesting end in itself, rather than just a secondary means for solving the clicking task. Admittedly, it is difficult to objectively verify our intuitions.

**Figure 6:**
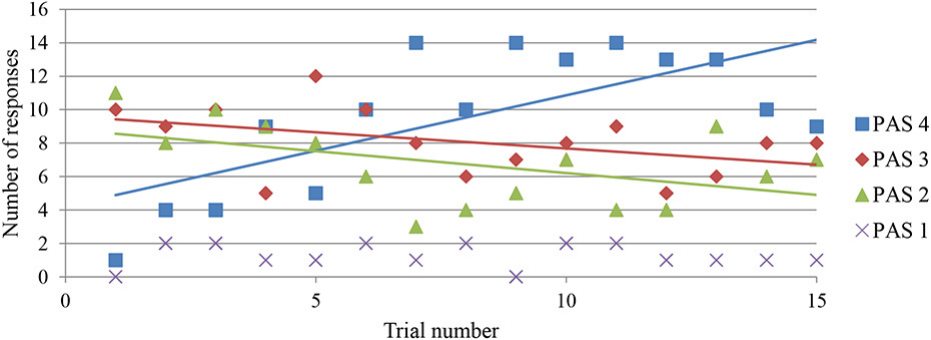
**Changes in the modified Perceptual Awareness Scale (PAS) ratings over the sequence of 15 trials**. After each trial in which they clicked, players were asked to give a PAS rating between 1 and 4: “Please select a category to describe how clearly you experienced your partner at the time you clicked: (1) No experience, (2) Vague impression, (3) Almost clear experience, (4) Clear experience.”

As a first step toward a personal-level explanation for the tendency of increasing joint clicking delays, we can evaluate whether there are corresponding qualitative changes in the participants' experience. As shown in Figure 6, there does indeed seem to be a correlated change in the reported clarity with which the other's presence is perceived. While low to medium levels of clarity predominate during the first few trials, there is an increase in the number of reports of maximum clarity until these reports come to predominate. Given that clicks in Single Success trials are most frequently associated with low to medium levels of clarity (see Figure 5 in Froese et al., 2014), this suggests that we are actually dealing with a qualitative change in the kind of mutual interaction that players engage in. Their engagements develop not only to be longer, as suggested by the increase in Joint Success clicking delays, but also more clearly social.

We expected that the nature of this qualitative shift in interaction had something to do with the emergence of more structured forms of co-regulated interactions, especially cases of turn-taking and mutual imitation. However, applying the objective measure of turn-taking described in our original study (see Supplementary Information Section S3 for details), which we had used to demonstrate that clearer experiences of the other player are preceded by more pronounced turn-taking interaction, did not reveal a very remarkable upward trend when viewed across trials, at least not when we average the turn-taking measure across all 17 teams (Figure S6). It may be that this measure is too crude to detect an increase in the co-regulation of interaction. And it is also possible that there are no general trends in turn-taking across teams; pairwise developments of mutual interaction may be too idiosyncratic for such averaging to be meaningful.

**Figure 7:**
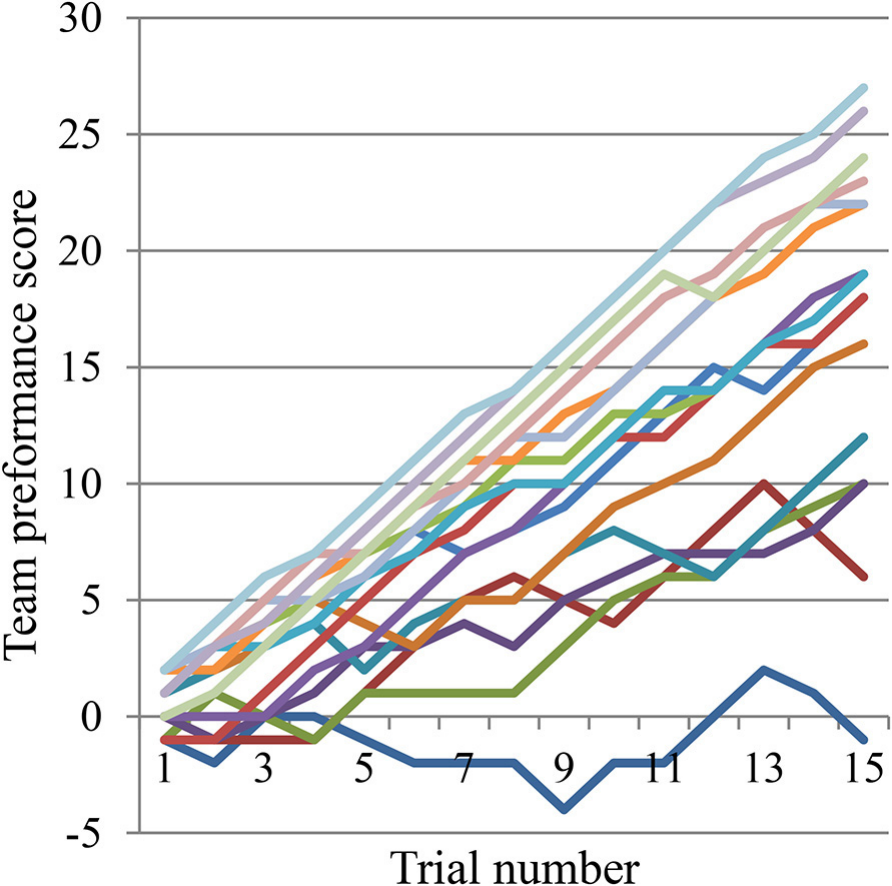
**Changes in team performances over 15 trials**. In each trial a player can make a correct click (+1 point), a wrong click (−1 point), or no click (no change). The final maximum possible team score is 30 (15 trials × 2 correct clicks).

**Figure 8:**
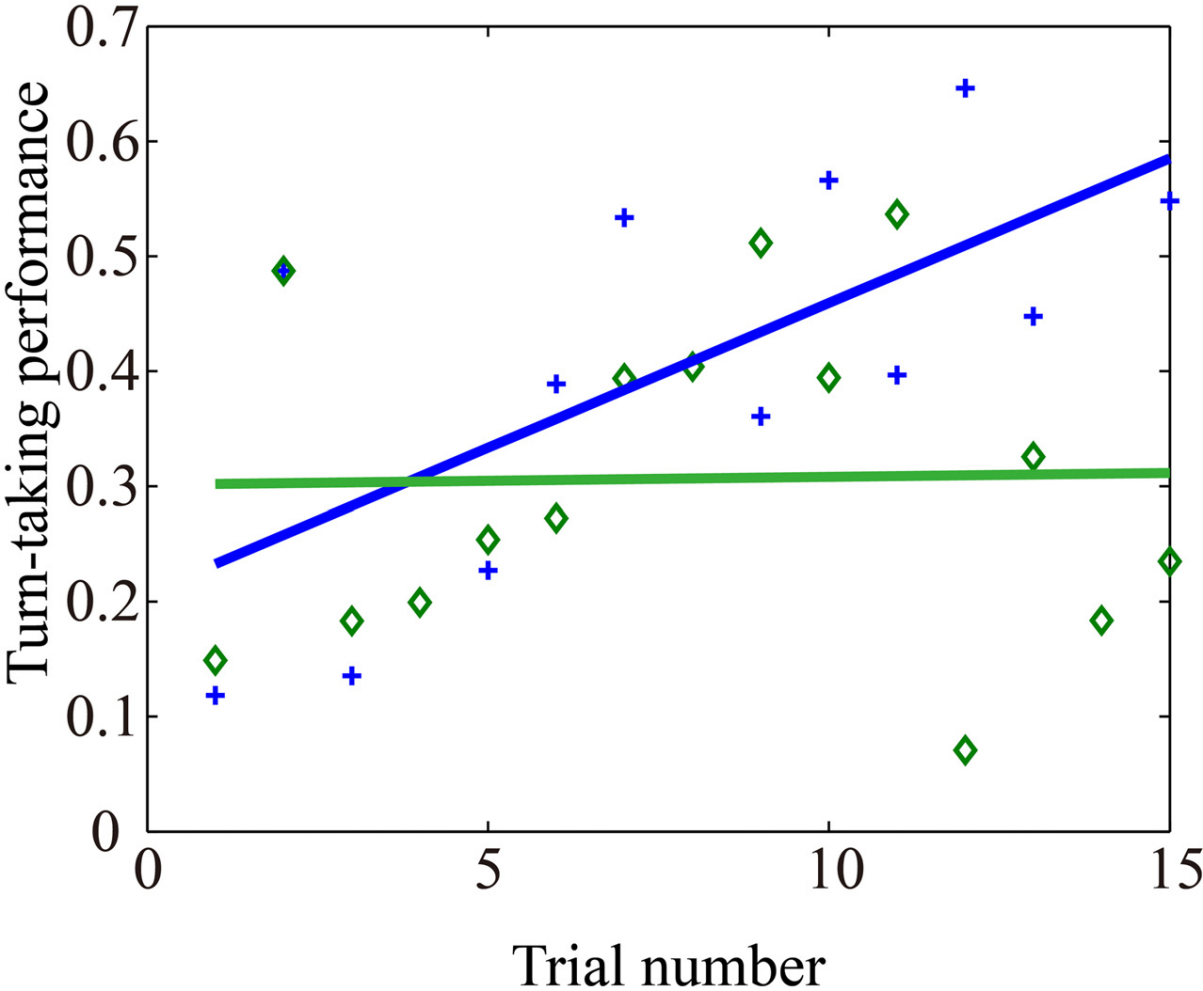
**Changes in amount of turn-taking of best team over 15 trials**. For each correct click in each trial we calculated the amount of turn-taking that had taken place between the players during the preceding 10 s (range [0, 1]). Crosses and diamonds represent turn-taking before correct clicks by Pa and Pb, respectively. This team (E14) managed to score 27 points (see top line in Figure 7).

The second possibility is supported by a comparison of developments in each team's clicking performance, which reveals that there are indeed different clusters of expertise (Figure 7). Future research may therefore be better served by focusing the diachronic analyses on selected teams. For instance, if we examine the changes in turn-taking performance of the best team alone we do find a notable upward trend over time, which remains consistent at least for one of the players (Figure 8). This is not the only case with such an upward trend but, as already indicated by Figure S6, it cannot be generalized. Many trials show no discernable trend, and there is even an example of a downward trend. Moreover, even this exemplary best case shows that the regular turn-taking interactions that had slowly been established during the first half of trials loose some of their regularity during the last 5 trials.

We note that this kind of transition is consistent with the findings of research in dialogic joint activity: “since one of the hallmarks of coordinated dialogue is its progressivity, the development of procedural coordination necessarily involves the *differentiation* of interlocutors' turns as coordination increases” (Mills, 2014, pp. 161–162). This increasing differentiation may also help to account for the facts that players click more conservatively during the second half of trials, and that they click in a less synchronized manner. As players implicitly learn how to co-regulate their interaction, simple interactive synchrony changes into more complex interpersonal synergy (Fusaroli et al., 2014).

### Evidence for developmental stages of social awareness

Following Reddy's (2003) work on developmental psychology, we hypothesized that participants' social awareness emerges in situations of mutual attention, in which one's awareness of the other's presence is first framed in terms of the other's attention to one's self in general, followed by mutual attention to what one's self specifically does. We did not consider the further progression to triadic joint attention.

An initial evaluation of participants' first-person reports suggested the possibility that there could be two distinct forms of awareness of being the object of the other's attention, namely depending on whether this awareness is mutually shared or not. In some cases people described awareness of being the object of the other's attention, but without thematizing the other's awareness of this awareness. Such descriptions of an individualistic awareness of being the other's object of attention may simply be a consequence of the technical specificities of the perceptual crossing setup. An actively searching participant cannot in principle distinguish between a completely immobile (or nonresponsive) participant and the static (or shadow) object. This means that there is a possibility of one participant having awareness of the other's attending presence, but without the other sharing in that awareness of attention.

Nevertheless, we highlight that analogous situations exist in human development. As Tronick discusses at length, a newborn lacks control over its own movements to the point that “what he is doing is messy – variable, unstable, disorganized” (2004, p. 307). And Reddy considers non-responsiveness to be an intentional action with which infants sometimes counter being the object of other's attention: “Infants can also be indifferent to others' visual attention, as anyone knows who, trying to engage a 2-month-old, has had the infant glance expressionlessly at them and turn away” (2005, p. 97). We can also consider cases of pathological development. For example, Tronick (2004, p. 304) examines the pathological apathy that is exhibited by chronically deprived orphans. When attending to such individuals we may remain unaware of the extent of their awareness of being the object of our attention, even though they might actually be aware of our attending presence.

We therefore defined three categories of experiencing the other's presence, which incrementally build on each other: (A) individual awareness of being the object of the other's attention, (B) mutual awareness of being each other's objects of attention, and (C) mutual awareness of specific aspects of the interaction being the object of joint attention. The categories overlap to some extent, but essentially category A includes only reports of awareness of the other's self-directed actions, B additionally required awareness of mutually responsive interaction, and C additionally required awareness of joint attention on something specific other than the selves, for example an arbitrary pattern of mutual contacts that had acquired special communicative significance.

After each trial, participants could write as little or as much as they wanted within 2 min until the next trial started. The questionnaire sheet asked them to describe the sensation of having encountered the other at the time of the click, and more generally to describe the strategy they had used during the trial. There were 472 instances of a participant having voluntarily written at least some text after a trial. Mostly these were fragmentary statements, with only very few responses consisting of several sentences.

Each of these responses was coded as belonging to one of the three social awareness categories (A, B, or C) or not assigned to a category (N/A). It was quite a challenge to categorize the responses. Wherever possible we based our categorizations not only on the brief description of the experience, but also on the brief description of the strategy, as well as descriptions provided for preceding and subsequent trials (e.g., participants often abbreviated by writing “same as above”). In cases where different categories where implied by a description of an experience compared to the stated strategy, for example if a participant only reported having individual awareness of being the object of the other's attention although an interactive strategy was described, the category of the experience took precedence. In order to get an estimate of interobserver reliability, two of us (TF and HI) independently did the coding. The results are shown in Table 1.

**Table 1:**
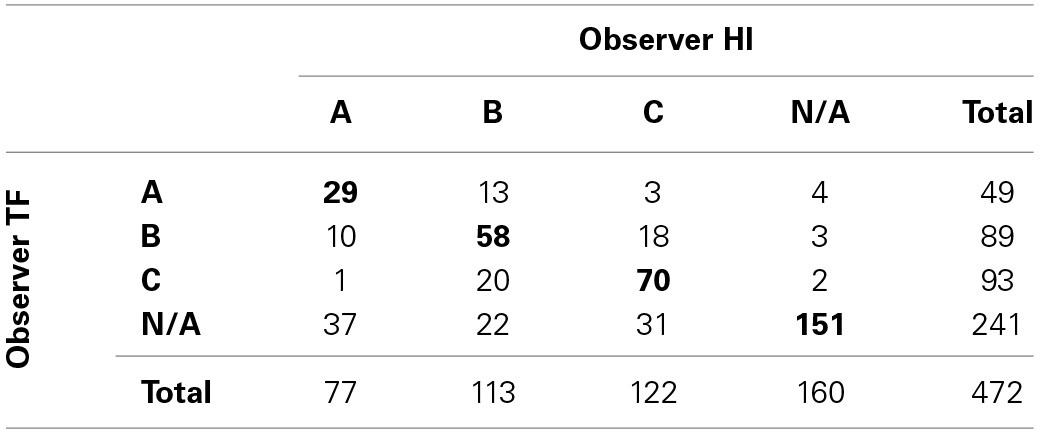
**Coding of free-text responses**.

*There were 510 opportunities for giving free-text responses (15 trials × 17 teams × 2 players), out of which 472 resulted in some written text. Two experimenters went through these responses with the aim of coding each into one of three categories: ***(A)*** individual awareness of being the object of the other's attention, ***(B)*** mutual awareness of being each other's objects of attention, and ***(C)*** mutual awareness of specific aspects of the interaction being the object of joint attention. If no category was applicable or there wasn't sufficient text to make a decision, the response was coded as N/A. Bold numbers represent the number of responses for which the coders were in agreement*.

In total there were 308 coding agreements, which is 65% of all responses. Given the frequency distribution of the four types of codings (A, B, C or N/A), the expected percentage of agreement is 29%. This gives an interobserver-reliability kappa of 0.51 (see Supplementary Material Section S3 for calculations), which can be interpreted as moderate agreement. Given the sparse responses collected during the original study, this is probably all that can be hoped for at this point. Moreover, it is encouraging that disagreements tended to occur more frequently between consecutive stages of awareness (i.e., between A and B, or B and C, rather than A and C), which is to be expected given that the three categories build on each other. In the following we limit our analysis to only those responses for which there was an agreement between the two coders. First, in order to illustrate how people responded and how we coded, we provide 10 examples for each of the three categories in Tables 2–**4**, respectively.

**Table 2:** **Category A: individual awareness of being the object of the other's attention**.

**Trial ID**	**First-person report**
E3T1Pa	*He touched me* periodically.
E15T13Pb	I felt that the partner *actively searched for me*.
E3T4Pb	I feel his *searching me*.
E7T9Pb	Again, I felt *being inspected* - an object was moving back and forth *across me*.
E6T10Pb	I encountered it at close positions. I am not sure but it tried to *survey me*.
E2T1Pa	Its movements didn't seem random or recorded. He was trying to *find me*. He was moving while staying on the same area.
E14T3Pa	I felt that the partner *checked if I* was the static object.
E1T11Pb	She seemed to *look for me* and always *come closer to me*.
E1T5Pa	The partner's avatar was again *moving around my avatar*.
E6T15Pa	I thought the partner *wander around my position* for a while after *touching me*.

*Ten exemplary first-person reports (emphasis added)*.

**Table 3:** **Category B: mutual awareness of being each other's objects of attention**.

**Trial ID**	**First-person report**
E14T1Pa	I felt that it *chased me*.
E14T2Pb	I felt that the partner was *leading me*.
E5T13Pa	I felt I *met with* my partner.
E6T14Pb	It stopped once *to see how I react*. After moving a bit, it came to me. Probably it was the partner.
E3T12Pa	Touched *each other*.
E1T9Pb	She likes me!
E7T3Pb	Thought we're *contacting each other*.
E11T8Pa	It *followed me* when I ran away.
E11T8Pb	I had an impression that *we are interacting* for a long time.
E7T11Pb	Felt like *crossing each other*.

*Ten exemplary first-person reports (emphasis added)*.

**Table 4:** **Category C: mutual awareness of specific aspects of the interaction being the object of joint attention**.

**Trial ID**	**First-person report**
E2T10Pa	We exchanged *patterns* so it was clear it was him.
E2T10Pb	Found object that responded to my *taataa, stop, taa-taa*, etc.
E1T9Pa	I think there was a *turn-taking*-like communication.
E1T10Pa	It looks like we've established a way to *communicate*.
E10T14Pb	The *rhythm* of touching was alternately exchanged.
E11T3Pb	I made “*zu-zu-zu*” by moving left and right and stopped. The partner moved in the same way and I felt “*zu- zu- zu-”*
E11T11Pa	I thought we made a *conversation*.
E17T13Pa	It did not cling but I felt that the partner sent the *“zu-zu” signal*.
E16T11Pb	The partner synchronized with *time gaps* that I sent.
E14T4Pa	I felt the partner *imitated* me.

*Ten exemplary first-person reports (emphasis added)*.

TF and HI jointly classified 29, 58, and 70 responses as belonging to categories A, B, C, respectively. Given that these three categories can be interpreted as analogous to the first stages in the development of social awareness, from passive individuality to active mutuality to co-regulated triangulation on a third element, we expected there to be a corresponding increase in the reported clarity of perceiving the other's presence. Or to put it differently, following the hypothesis formulated by Froese and Di Paolo (2011), we expect there to be a correlation between the extent of co-regulation and the sense of sociality in the experience. The increasing number of reports found for each category already suggests this trend, since having a clearer experience of the other makes it easier to report it. And we further confirmed this hypothesis by evaluating the perceptual awareness scale (PAS) ratings associated with each category.

In order to determine if there was a significant difference between the average PAS ratings reported for the categories we applied one-tailed, two-sample equal variance *t*-tests. There were 24, 56, and 68 PAS scores associated with the agreed categories A, B, and C, respectively. The equality of variances was verified using an *f*-test for each comparison. The average reported clarity of experiencing the other's presence for category B experiences was slightly but not significantly higher than for category A (μ_A_ = 2.83; μ_B_ = 3.05; *P* = 0.15), but the average clarity for category C was significantly higher than for category B (μ_C_ = 3.62; *P* = 3.71 ×10^−6^).

The fact that the clarity of social awareness associated with categories A and B was not significantly different suggests that these categories may not be experienced as qualitatively distinct situations from the first-person perspective. This is consistent with Reddy's (2003) approach, which does not allow for a purely individualistic awareness of being the object of the other's attention but treats such awareness as always already mutual to some extent. Indeed, from what we have observed while running the study, it does seem highly unusual for participants to remain completely nonresponsive while being their partner's object of attention. Typically, after having received a few touches the subjects of attention quickly get pulled into a mutually responsive interaction. In the following we therefore collapse categories A and B into a single category of mutual awareness, category AB.

**Figure 9:**
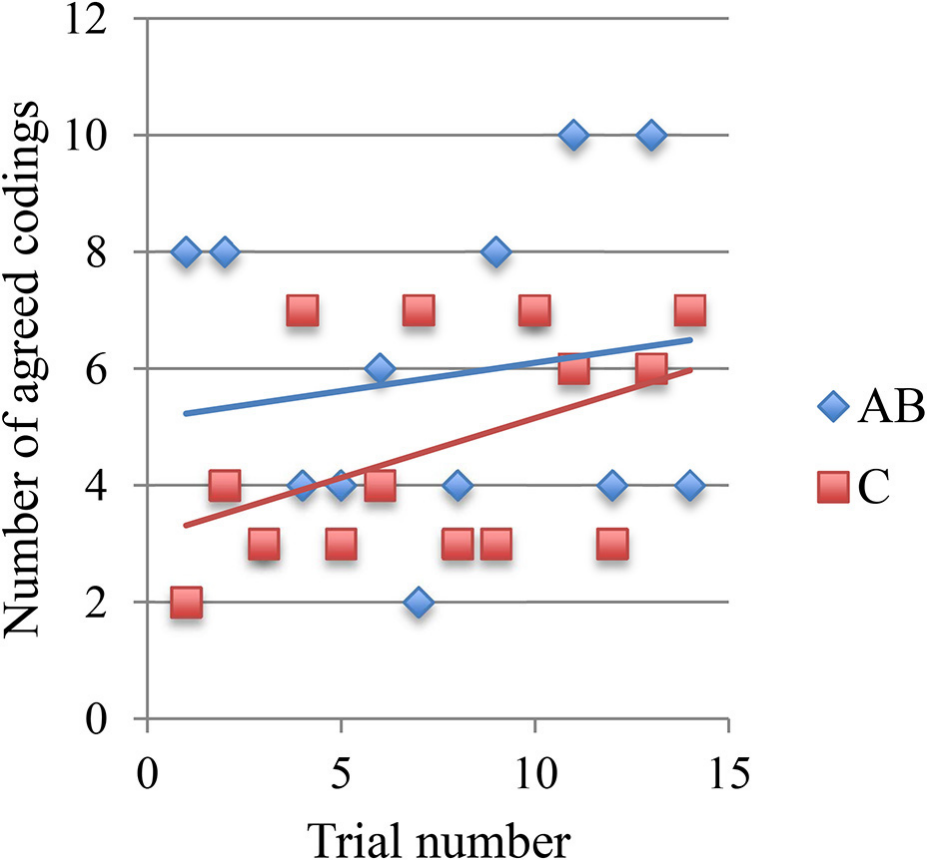
**Changes in how participants described their social awareness**. Categorizations were based on brief free-text first-person reports. Only cases where both coders agreed were considered. Category AB: mutual awareness of being each other's object of attention (combining categories A and B). Category C: mutual awareness of joint attention to aspects of the interaction.

The final step of our analysis was to determine if experiences belonging to categories AB and C actually followed a sequence. Given the developmental sequence from AB to C, we expected responses categorized as AB to be more frequent than C during the initial trials. We may also expect that category C is more frequent during later trials, although it does not necessarily have to displace category AB since C can be seen as a more specific articulation of AB. These predictions are partially supported by the data (Figure 9). In the first couple of trials there are indeed more cases of AB than C. The frequency of C tends to increase over the subsequent trials, but it never fully becomes the dominant category. These findings are suggestive, but the trends are not that well pronounced and may be biased by the small sample size.

Clearly, a proper evaluation of our hypothesis that the developmental stages of social awareness can be recapitulated using this kind of experimental setup requires a more systematic collection and analysis of subjective reports. Developmental studies using the microgenetic method have long emphasized the need for dense observations of individual cases (Siegler and Crowley, 1991). Due to the limited number of usable free-text responses, and even less agreed upon codings, we averaged categorizations across all 17 teams, which may have further obscured any idiosyncratic team-based trends. Nevertheless, these tentative results at least hold out the prospect that more distinguishable developmental trends in social awareness could be discovered by studies that are specially designed to elicit detailed first-person reports.

Participants could also be phenomenologically trained beforehand to become more aware of their different kinds of experience (Lutz, 2002). Another possibility is to interview them about their experience using a specialized method (e.g., Petitmengin, 2006; Hurlburt, 2011). Biases associated with experimenters' classifications of the written reports could be avoided by asking participants to define and select categories that best describe their own experiences (Lutz et al., 2002).

## Discussion

We have proposed that a suitably implemented perceptual crossing paradigm can fill a gap in experimental psychology. Following enactive theory, we hypothesized that we should find something akin to the main stages of development of social awareness in infants recapitulated in adults if they are forced to implicitly relearn the skill of social perception. A sequence of three categories was defined: (A) individual awareness of being the object of the other's attention, (B) mutual awareness of being each other's objects of attention, and (C) mutual awareness of specific aspects of the interaction being the object of joint attention. The preliminary results we have presented suggest that our hypothesis has merit, although the methods still need refinement. We found that there was an average increase in reported clarity of social awareness over trials, but it is challenging to find an objective explanation of this phenomenon. Turn-taking is only partially responsible, and a team-based measure may be more appropriate. We also found that there is no significant difference between categories A and B in terms of the associated clarity of social awareness, with only C being significantly clearer, which we argued is in line with Reddy's (2003) original proposal, although evidently more precise phenomenological work is needed. At least these diverse and complex results already have the benefit of warning us against idealizing the phenomenon of development as a linear sequence of independent stages.

Although it was difficult to find general trends across all teams, many participants were able to sense the other's attention to their objective presence, and to engage in co-regulated interactions that involved mutual attention, such as feeling being chased/being led (see Table 3, rows 1 and 2, for descriptions by one team). Some participants were able to further develop these co-regulated engagements into communication games involving turn-taking and mutual imitation, such as passing patterns of activity between each other (see Table 4, rows 1 and 2, for reports by one team). On the basis of such coordinated interactions apparently it even became possible to perceive the other's emotional state across the HCI, as predicted by Lenay (2010).

For example, after trial 10 one player somewhat confidently remarks: “I think I am pretty sure that I could communicate about my intention” (E10T10Pa), and two trials later he writes: “Same as before, but I felt that the partner is anxious” (E10T12Pa). Did this player correctly make sense of his partner's emotional state of mind? Given the unfortunate scarcity of free-text descriptions that were generated by the original experiment, it was usually impossible to evaluate this kind of question. However, here we happened to be lucky because after the next trial his partner writes: “I think my click was correct but if this response was autonomous object's, I will get anxious” (E10T13Pb). In other words, despite the extreme poverty of the stimulus provided by this minimal HCI, namely a sequence of binary (on/off) tactile sensations, one player seems to have correctly noticed some anxiety in the other's style of engagement.

This finding is consistent with studies showing our propensity to discern intentional states on the basis of minimal movement information (Blake and Shiffrar, 2007), such as detecting other people's emotions from the point-light displays of their dances (Brownlow et al., 1997). It is still debated if this ability is best explained as a direct perception of the other's intentional state in their behavioral expression (Stout, 2012), or if perception just presents us with meaningless “surface behavior” that needs to be cognitively penetrated to gain access to the underlying intentions (Baldwin and Baird, 2001). We suggest that with this experimental setup social understanding might be productively analyzed as a case of direct perception by interaction (De Jaegher, 2009). Due to the constraints of the HCI it is impossible to discern the other's intentions without at the same time interacting with them, and this interaction can evoke a felt sensation of the other's mental state. Using the terminology introduced to developmental psychology by Stern (1998), we can describe this encounter as an amodal perception of the other's vitality affect in the activation contours traced by their movements-in-interaction. The result is a felt impression of the other's state, e.g. “She likes me!” (E1T9Pb), which in turn will modulate the perceiver's expression, thereby making an impression on the other, and so forth. Interacting players are thereby able to create an intertwinement of embodied affectivity, which is a form of embodied communication (Fuchs and Koch, 2014). Movement and being moved both have spatial and emotional components.

From this perspective we can also better understand why a player would terminate an interaction that is too repetitive and “mechanical” (see Figure 4C). The other player might keep faithfully replicating an already established signal, but without at the same time allowing their movement to resonate with the other's changing expressions they fail to participate in a shared affective space. When the signal stops being grounded in a mutually affecting situation it looses its communicative value; it becomes an empty form that obscures rather than expresses the other's subjective presence. This example nicely shows the primacy of embodied communication via interbodily resonance, in contrast to the traditional starting premise of sending and receiving symbolic signals across pre-defined channels. The importance of common ground for the emergence of an embodied communication system has been observed before (Scott-Phillips et al., 2009). Here we saw that it continues to be crucial even after the establishment of that system in the interactive maintenance of its meaningfulness.

However, we acknowledge that this interactive-perceptual strategy is not the only way of realizing the task of locating the partner. As we discussed previously (Froese et al., 2014), one outstanding participant managed to get nearly perfect clicking scores while never reporting any direct perceptual awareness of the other's presence. Leaving the free-text boxes asking for descriptions of his felt sensations entirely blank, he only provides a few statements of his strategy that reveal the perspective of a detached observer: “Because the partner generated intermittent stimulation, I also reply the intermittent stimulation” (E15T2Pa). Similarly, another participant insisted on relying more on an individualist cognitive strategy: “Felt like it was him. But every time I say feel, I must say I rely much more on thinking about my strategy and sticking to it.” (E2T14Pb). But then again, the fact that at least some more cognitivist strategies were employed is not all that surprising. After all, participants were adults who in real life already had fully developed social skills and who were confronted with a breakdown of these skills, a breakdown that could be expected to elicit more reflective awareness and cognitive compensatory strategies (Dreyfus, 1991).

We note that accepting the importance of the individual is not a problem for this framework because the interactive turn in cognitive science is not a return to the old days of behaviorism or some kind of extremist externalism. The internal organization of agents is a central concern of the enactive approach to social cognition (Froese and Di Paolo, 2011). Neither is this concession to the individual and its internal milieu a return to the classical internalism of cognitivism, since all behavior is conceived of as a dynamical property of embodied and situated minds (Beer, 2000). The perceptual crossing paradigm thus provides a platform for gaining a better understand of the diversity of individual and interactive styles that exist. These differences were mainly ignored in the current analysis because we were looking for statistically significant trends that were averaged over players and teams.

Apart from confirming the preliminary results presented here, it would be interesting to use this approach to investigate other hypotheses about the development of social awareness. For instance, Stern (1998, pp. 56,57) assigns developmental primacy to amodal perception of the other's vitality affects over modal perception of overt acts and objects. Future work could attempt to use the current approach to study the developmental trajectory from the former to the latter. In addition, studies have found differences in phenomenology between people from an Eastern and Western cultural background, including divergences in their development of social experiences (Cohen et al., 2007). Although our study included participants from these two backgrounds, we did not distinguish between these groups. Conducting a between-group experiment might reveal differences in their development of social awareness. Finally, it is an interesting open question whether it is possible to modify the perceptual crossing paradigm so as to allow for the emergence of secondary intersubjectivity (Trevarthen and Hubley, 1978), including the triangulation of joint attention on external objects, which is predicted to follow after the stages of mutual awareness that we have described here (Reddy, 2005).

### Conflict of interest statement

The authors declare that the research was conducted in the absence of any commercial or financial relationships that could be construed as a potential conflict of interest.

## Footnotes

^1^In the category of mediating system we may also include cultural modulators of experience such as language (Bottineau, 2010), norms (Merritt, 2014), and institutions (Gallagher, 2013).

^2^At least this is the case for people who do not suffer from a mental disorder or some other unusual condition. For example, people with schizophrenia or with an autism-spectrum disorder tend to lack the capacity for direct social perception (Froese et al., 2013). Yet if our hypothesis were correct, it would suggest the intriguing possibility that this perceptual capacity could be partially recovered by engaging in some form of embodied practice that enhances skillful engagement with others, which is consistent with the aims of movement therapies (Fuchs and Koch, 2014).

^3^Some developmental psychologists have even argued that, just like ontogeny was supposed to recapitulate phylogeny (i.e., Haeckel's biogenetic law), there are important parallels between cognitive development and the history of science (for a critical discussion of Piaget's theory, see Franco and Colinvaux-De-Dominguez, 1992). Here we restrict ourselves to comparing two processes of change that can take place within the time scale of one individual's lifetime.

^4^There are two reasons for increased conservatism of clicks compared to the study by Auvray et al. (2009). First, players were allowed to click maximally only once per trial rather than as many times as they wished. Second, the experiment was run as a team-based tournament game and a wrong click meant loss of a point for the team (a correct click gained the pair of participants a point, whereas no click left the score unchanged).

^5^This code uniquely identifies the first-person report. In this case it was during (E)xperiment 18, after (T)rial 3, and written by (P)layer ‘b’. Since the original perceptual crossing experiment was conducted at the University of Tokyo, many first-person reports were originally written in Japanese, including those of experiment 18 discussed here. They were translated into English by HI. The number of experiments goes to 18, even though there were only 17 teams, because the numbering includes a test experiment (E4) between TF and TI that was removed from the analysis.

^6^Note that the number of Wrong Clicks is not identical with the number of Wrong Click trials, because there were 1, 1, 1, and 2 trials with jointly wrong clicks in trial numbers 5, 9, 14, and 15, respectively.
