# Supplementary material for: Using minimal human-computer interfaces for studying the interactive development of social awareness
Source: Front Psychol. 2014 Sep 26;5:1061. doi: 10.3389/fpsyg.2014.01061 (PMC4176033; doi:10.3389/fpsyg.2014.01061)
Supplement: Supplementary file 1 [file DataSheet1.DOCX]

**Supplementary information**

**Using minimal human-computer interfaces for studying the interactive development of social awareness**

Tom Froese, Hiroyuki Iizuka, and Takashi Ikegami

This supplementary information consists of three sections. Section S1 describes in some detail the tests we did in order to examine the influence of a potential confound with respect to making a diachronic analysis of our original perceptual crossing study. In section S2 we include a couple of figures which further illustrate the results that are described in the main text of the paper. Section S3 provides details about how we calculated the interobserver reliability of categorizing first-person reports, and the turn-taking performance.

**S1. Analysis of influence of initial distance between avatars and static objects**

One confounding factor in our results is that we did not randomize the initial positions of players’ avatars across teams. In other words, each trial featured a different random starting configuration, but this sequence of configurations was the same across all 17 teams. It therefore cannot be ruled out completely that the developmental trends we found across trials were to some extent influenced by confounding properties of the starting configurations. Nevertheless, as we try indicate with the following figures, it seems reasonable that the increase in clicking performance over trials had more to do with implicit learning and actual improvement of social skill and social perception rather than with trial configurations becoming progressively easier.

The initial configurations can be characterized by two essential factors, namely (a) the avatars’ initial distances from their respective static object, and (b) the avatars’ initial distance from each other, which is directly proportional to the avatars’ initial distance from each other’s shadow object. We will consider these two factors in turn.

On the one hand, it might be expected that there is a relationship between the number of clicks on static objects and the initial absolute distance between a player’s avatar and its static object. But when we plot the number of clicks against starting distance, there is only a weak relationship (Figure S1). On the other hand, it could also be that the influence of the initial distance to static objects is more subtle, namely as a source of noise and interference during avatar-to-avatar interactions during the initial period of a trial. However, again there is only a weak relationship at best.

**FIGURE S1 | Relationship between the frequency of object clicks with respect to initial absolute distance between a player’s avatar and its static object.** The actual starting range [-300, 300] was converted to range [0, 300] because the maximum absolute distance between two positions is 300 (since the endpoints of the virtual 1D environment connect). There are 30 distance measurements (i.e., 15 trials for each of the two static objects). The maximum number of possible clicks is 17 (i.e., when all 17 players paired with that static object click on it during a trial).

Another way of evaluating the potential extent of this unwanted influence is to check if there was a significant increase of initial absolute distance to static objects across the sequence of trials, and whether this increase could explain the increase in the rate of successful clicks. In Figure S2 we plot the initial absolute distance averaged over both players and the total number of clicks on avatar by both players (multiplied by 10 for purposes of easier comparison) over the 15 trials. As indicated by the linear trend lines, the average initial distance to static objects does not decrease significantly across trials. The trend of total clicks on the other’s avatar, on the other hand, shows a notably positive gradient. Initial distance to static objects is therefore not a factor that needs to be considered when explaining increasing clicking success.

**FIGURE S2 | Plot of total clicks on avatar and average initial absolute distance between players’ avatars and their static objects over the sequence of 15 trials.** The number of clicks was multiplied by a magnitude of 10 to facilitate comparison with the distance measurements. While the fluctuations of initial distance conserve their average over trials, as expected since they are drawn from a random distribution, the number of successful clicks increases.

However, it could also be argued that the initial distance between avatars themselves could have influenced the diachronic results presented in our paper. In particular, it might be expected that there is an inverse relationship between the number of avatar clicks and the absolute distance between avatar starting points, such that players are more likely to click on each other when they start a trial nearby each other. In order to evaluate this possible confound we plotted the number of clicks for all object types with respect to the initial absolute distance between avatars (Figure S3). There seems to be weakly positive correlation between starting closeness and number of successful clicks, which could have influenced the results. In particular, there may be a positive effect of very small distances (< 30) on the number of avatar clicks, but the largest number of successes was actually achieved after starting from far away (ca. 207).

**FIGURE S3 | Relationship between the number of object clicks with respect to initial absolute distance between players’ avatars.** The actual starting range [-300, 300] was converted to range [0, 300] because the maximum absolute distance between two positions is 300 (since the endpoints of the virtual 1D environment connect). There are 15 measurements (i.e., 15 trials with different initial avatar starting configurations). The number of possible clicks for each distance is 34 (i.e. when both players of all the 17 teams click on the same type of object during a trial).

To evaluate the extent of this positive influence on developmental trends we can look at the initial distance measures and clicking successes across trials (Figure S4). There does seem to be a notable tendency for the distance between avatar starting positions to decrease over trials, while clicking successes increase. This could support the idea that the tendency toward increasing number of clicks on the other’s avatar is at least partially explainable in terms of the effects of decreasing mutual initial distance rather than implicit learning. On the other hand, when looking at the scatter plots in more detail, the relationship between mutual distance and clicking success does not seem to be very deterministic, since the best overall score of 29 avatar clicks occurred during trial 10, which had one of the most distant initial configurations (ca. 207). Out of the two potential confounds, namely starting distance to the two static objects and starting distance between avatars, it is the latter that seems to be more problematic.

**FIGURE S4 | Plot of total clicks on avatar and initial absolute distance between players’ avatars over the sequence of 15 trials.** To facilitate comparison with the distance measurements, the number of clicks was multiplied by ten. The linear trend lines show that while players’ mutual initial distance decreased on average, the number of clicking successes increased. This suggests that there may have been some influence of shorter mutual initial distances on clicking success.

Yet players are not bound to the initial configuration because they can move for up to one minute, which is time enough to repeatedly traverse the virtual 1D environment. We therefore expect that the effects of close initial distances should be marginalized by the subsequent interaction dynamics. This prediction is indirectly confirmed when considering trends in the delays between correct clicks across trials (Figure S5). If the decreasing initial distance over trials has a facilitating influence on the increasing number of correct clicks, then we should expect delays between the two correct clicks of Joint Success trials to decrease over trials. In other words, since the closer initial distances should presumably help players to find each other more rapidly, they should both be expected to click more rapidly, too. However, precisely the opposite tendency can be observed in the figure: shorter delays tend to become less frequent over trials while longer delays become notably more frequent.

In summary, the effects of initial configurations are likely to be largely negligible for our diachronic analysis. Nevertheless, due to the experimental design of the original study, some form of configuration-based confound cannot be ruled out completely from the results. In particular, decreasing initial mutual distance could have played a role in facilitating increasing clicking successes. Accordingly, the results we present in the paper must be taken as preliminary. It is evident that future studies should also randomize starting positions for each trial across teams in order to cancel out potential influences of spatial configurations on developmental trends.

**S2. Supplementary figures**

In this supplementary section we include two additional figures that help to illustrate the arguments made in the main text and in supplementary text above.

**Figure S5 | Changing frequency of delay ranges between clicks in Joint Success trials.** Most jointly successful clicks occur within seconds of each other (0 to 10 s), especially during the first half of trials. However, while the total number of Joint Success cases increases over trials, this is largely due to an increase of cases with longer delays between clicks (10 to 60 s).


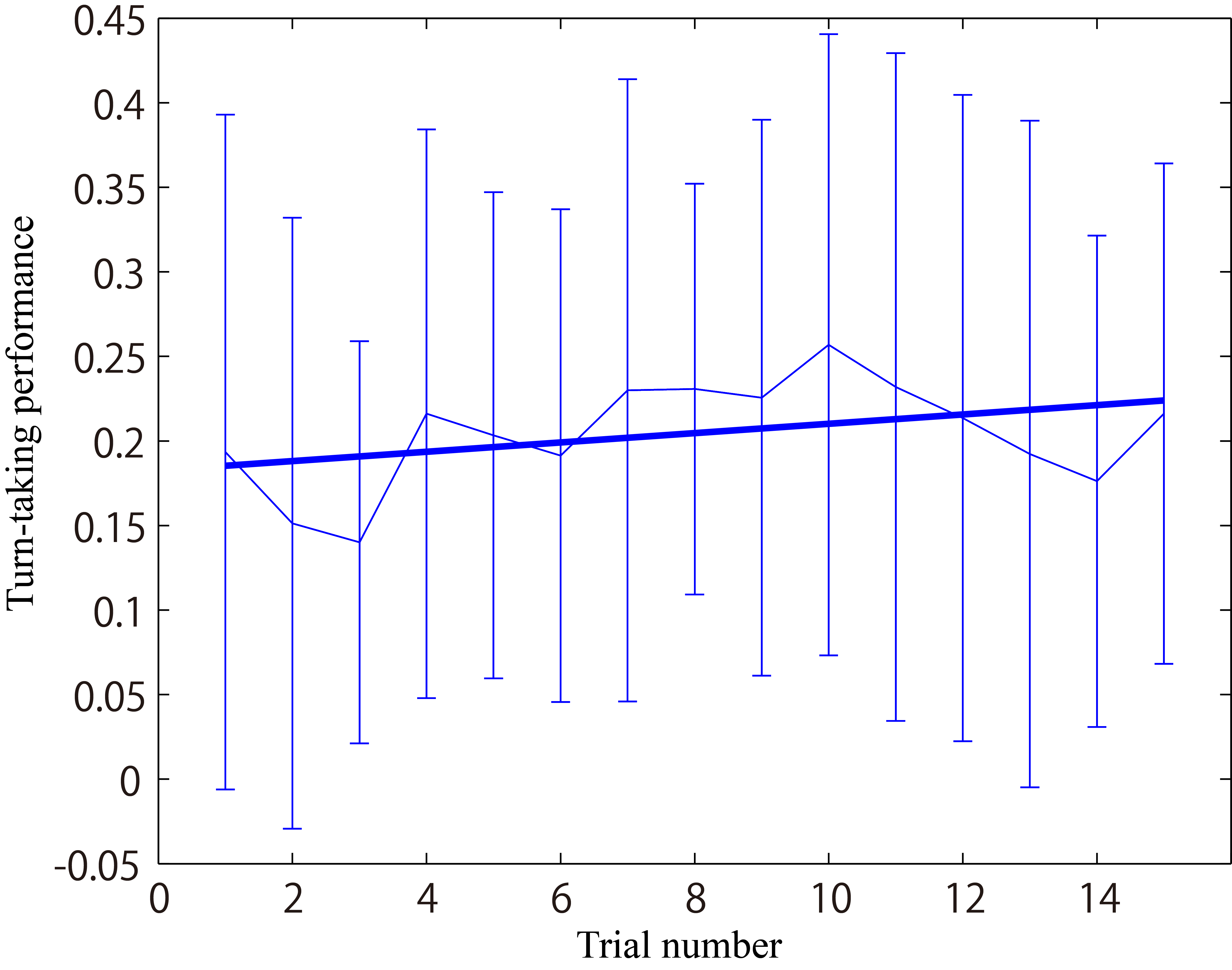


**Figure S6 | Changing prevalence of average turn-taking performance.** Turn-taking was measured during the 10 seconds preceding participants’ clicks. We applied the measurement method described in our original perceptual crossing study (for details of the calculation, see section S3.2 below). Error bars represent one standard deviation.

**S3. Supplementary equations**

S3.1 Calculating inter-observer reliability

Given the data shown in Table 1, we can calculate *observed* agreement as follows:

$$\frac{(29+58+70+151)}{472}=0.65$$

We can also calculate how much agreement would be expected by chance alone by taking into account the frequency of a categorization and using this to calculate the probability of both coders assigning that same category. The *expected* agreement is the sum of the union of both coders’ probabilities for each category.

$$\left( \frac{77}{472}\times\frac{49}{472} \right)+\left( \frac{113}{472}\times\frac{89}{472} \right)+\left( \frac{122}{472}\times\frac{93}{472} \right)+\left( \frac{160}{472}\times\frac{241}{472} \right)=0.29$$

These two percentages allow us to evaluate how different the observed agreement is from the expected agreement, for example by calculating the kappa value (Viera and Garrett, 2005). The kappa value has the standardized range [-1, 1], where 1 is taken to represent perfect agreement, 0 means that any agreement is only by chance, and -1 is perfect disagreement. Plugging our values into the standard equation yields:

$$\frac{\left( 0.65-0.29 \right)}{\left( 1-0.29 \right)}=0.51$$

According to Landis and Koch (1977), this kappa value falls into the category known as “moderate” agreement (ranging from 0.41 to 0.60). Since disagreements between A and C are more severe than disagreements between A and B or B and C, we can also calculate a weighted kappa based on the following table of weights.

|  | A | B | C | N/A |
| --- | --- | --- | --- | --- |
| A | 0 | 1 | 2 | 1 |
| B | 1 | 0 | 1 | 1 |
| C | 2 | 1 | 0 | 1 |
| N/A | 1 | 1 | 1 | 0 |

Larger weights are assigned to categories that are further apart; zeros are assigned to default agreements. Here we have given disagreements between categories A and C twice the weight of other disagreements, including compare to disagreements about whether to abstain from applying one of the three categories (i.e., N/A). The weighted kappa is calculated as 1 minus the sum of products of observed codings and weights, divided by the sum of products of the expected codings and weights.

In this case the weighted kappa value is 0.54, which is slightly larger than the non-weighted kappa (0.51), indicating that there are many disagreements that are not that severe (i.e., as would be between A and C). Nevertheless, the weighted kappa is not sufficiently larger to fall into the next category known as “substantial” agreement, which ranges from 0.61 to 0.80.

S3.2 Calculating turn-taking performance

As described in the report of the original experiment by Froese, Iizuka and Ikegami (2014), several decisions had to be made regarding the calculation of a player’s turn-taking (TT) performance. For readers’ convenience we repeat here their description of the measuring method that was employed.

Froese et al. wanted a measure of TT performance that would exclude situations in which both players were continuously moving (or not moving) at the same time, or in which only one of the two players was continuously moving while the other was continuously stationary. They adopted the following procedure.

At each time step they classified each of the two players’ behavior in binary terms as either moving (1) or non-moving (0) by evaluating their computer mouse movement (they referred to these behavior time series as B1 and B2 for player a and for player b, respectively). Movement was considered to have taken place whenever the change of position *dx* from one time step to the next was bigger than an 8th of the avatar’s body size (i.e., 4/8 **=** 0.5 so that if *dx* **>** 0.5, 1, else 0). Since players often engaged in micro-saccades during their ‘‘turn’’ of an interaction, they chose to set a lower limit to the duration of pauses so as not to end up with a series of meaningless microturns. Thus, they only set behaviors to 0 if there was no motion over at least 50 consecutive time steps (500 ms), otherwise behaviors remained set to 1.

In order to determine a difference *D* in activity, they applied the logical ‘‘Not-And’’ operator to these two time series (i.e., D **=** B1 Not-And B2). Then, they assigned to each player their active contribution *C* of this movement exchange by applying the logical ‘‘And’’ operator and summing the result (i.e., C1 **=** sum(B1 And D); C2 **=** sum(B2 And D)). The overall TT performance for a given time period (here set to 10 s) before a player’s click could then be calculated by multiplying the players’ active contributions and normalizing the outcome such that TT **=** 4 * C1 * C2/T^2^, where *T* is the number of time steps. The range of TT performance scores is therefore from 0 to 1, with 0 representing a complete absence of TT interactions and 1 representing a perfect exchange of activity and passivity between the two players.

**References**

Froese, T., Iizuka, H., and Ikegami, T. (2014). Embodied social interaction constitutes social cognition in pairs of humans: A minimalist virtual reality experiment. *Sci. Rep.* 4. doi: 10.1038/srep03672

Landis, J.R., and Koch, G.G. (1977). The measurement of observer agreement for categorical data. *Biometrics* 33**,** 159-174.

Viera, A.J., and Garrett, J.M. (2005). Understanding interobserver agreement: The kappa statistic. *Family Medicine* 37**,** 360-363.
